# Systematic review and pooled analysis of randomized controlled trials in countries of the Gulf Cooperation Council (GCC): Methods and quality assessment

**DOI:** 10.15537/smj.2023.44.4.20220664

**Published:** 2023-04

**Authors:** Khalid S. Alraddadi, Fayzah H. Al-Adwani, Rajaa M. Al-Raddadi, Sultan H. Alamri, Iman K. Ramadan, Ahmad A. Mirza

**Affiliations:** *From the Department of Primary Health Care (Alraddadi, Al-Adwani), National Guard Health Affairs, King Saud bin Abdulaziz University for Health Sciences; from the Department of Community Medicine (Al-Raddadi, Ramadan), and from the Department of Family Medicine (Alamri), Faculty of Medicine, King Abdulaziz University; from the Department of Otolaryngology–Head and Neck Surgery (Mirza), Faculty of Medicine in Rabigh, King Abdulaziz University, Jeddah, Kingdom of Saudi Arabia; from the Department of Community Medicine (Ramadan), Faculty of Medicine, Al-Azhar University, Cairo, Egypt; and from the Department of Otolaryngology–Head and Neck Surgery (Mirza), Temerty Faculty of Medicine, University of Toronto, Toronto, Canada.*

**Keywords:** Clinical Trial, systematic review, bias, Arab world

## Abstract

**Objectives:**

To describe variations in characteristics of randomized controlled trials conducted in the Gulf Cooperation Council (GCC) countries, and critically appraising the quality of design, conduct and analysis of the trials.

**Methods:**

We carried out a systematically comprehensive electronic search of articles published between 1990 and 2018 and indexed in several databases: i) MEDLINE/PubMed, ii) EMBASE, iii) Cochrane Central Register of Controlled Trials (CENTRAL), iv) ClinicalTrials.gov, and v) World Health Organization International Clinical Trials Registry Platform. We summarized the overall risk of bias present in all analyzed studies using the Cochrane Collaboration risk of bias tool (CCRBT).

**Results:**

A remarkable shift in numbers of publications from 2006 onwards was found. The largest number of publications were from Saudi Arabia and consisted of hospitals/clinics based studies. Lack of randomization was found in the majority of reports, and nearly three-fourth of the studies involved the use of intention-to-treat (ITT) principle. However, the proportion of adequately generated random sequence methods has increased yearly, and this increase accounted for a relatively large proportion over the latter half of the studied period (*p*<0.001), in contrast to the proportion of allocation concealment and blinding. Journal impact factor was significantly correlated with the quality of random sequence generation (r=0.145; *p*=0.014).

**Conclusion:**

The randomization methods have gained more attention over the last 3 decades. Secondly, Journal impact factor can serve as an indicator of randomization quality. To mitigate the large rate of overall high risk of bias in GCC studies, high-quality trials must be considered by ensuring adequate allocation concealment and blinding methods. **PROSPERO No. ID:** CRD42022310331

**A** systematic review that involves examination of several studies can be used by the researchers to address a specific research question. Researchers are expected to evaluate such studies in terms of whether it was or was not properly conducted (high versus low quality). A randomized controlled trial (RCT) is a study design that is recognized as an essential standard for evidence-based medicine (EBM), which forms the basis of decision-making concerning the health care for patients.^1,2^ Randomized controlled trials are also useful in minimizing bias, producing dependable results and generating efficacy and safety data; however, if such a trial is designed and conducted in poor methods, it can lead to the reporting of unreliable results.^1,3,4^ Conducting reviews on methodological quality of studies will be helpful to avoid such erroneous conclusions, thereby preventing the use of poor basis for clinical applications.^1,5^ The high prevalence of communicable and non-communicable diseases in the Arab World necessitates high-quality research (such as RCT).^6^ In the Arab Gulf states, the cases of non-communicable diseases and their risk factors were found to have increased over time.^7^ This finding suggests the need for experimental research, such as RCTs, to address this alarming concern.

The Cochrane Collaboration Risk of Bias Tool (CCRBT) is one of the widely utilized reliable tools used to assess the quality of RCTs, specifically evaluating the risk of bias in 7 domains, namely, random sequence generation, allocation concealment, blinding of participant and personnel, blinding of outcome assessors, incomplete outcome data, selective reporting, and other sources of bias.^8,9^ These domains are related to bias in terms of selection, detection, performance, reporting, or attrition.

Research conducted with poor quality design could produce bias, which can significantly affect the accuracy, validity, and reliability of its result. This bias could be related to selection, performance, detection, attrition, or reporting of the study which can be described as high risk, unclear risk, or low risk. The Gulf Cooperation Council (GCC) countries are developing countries, and they are part of the Arab states. They are located in the Arab Gulf region in which research has been rapidly gaining momentum and rapidly expanding. Saudi Arabia, for example, ranked second in the total number of publications amongst all Arab states; yet limited systematic evaluations of the current standard of RCTs are available.^10-12^ Our review addresses this gap in which the results may be useful as a reference for future researchers in their specific area to work on meticulously designed studies addressing communicable and non-communicable diseases, and also indirectly prevent future unwanted medical expenses.

In this review, we aimed to evaluate the risk of bias present in RCTs that can be useful for identification of high quality studies and providing information to future researchers on how to design and conduct high quality studies.^1,5^ Our objectives were to identify published RCTs conducted in the GCC countries (Saudi Arabia, Bahrain, United Arab Emirates, Kuwait, Oman, and Qatar), to describe variations in general characteristics of these RCTs, and to critically appraise the quality of design, conduct, and analysis of RCTs in these countries.

## Methods

### Protocol and registration

The study was reported according to The Preferred Reporting Items for Systematic reviews and Meta-Analyses 2020 (PRISMA 2020) to ensure that all recommended information is captured.^13^

### Criteria for study selection

The current review included all published (written in English language) RCTs that were retrievable from certain databases and conducted on humans in the 6 countries of GCC since 1990 up to year 2018. Qualitative and quantitative data were evaluated.

### Electronic search

Two independent reviewers conducted a systematically comprehensive electronic search of RCTs published in English language between 1990 and 2018 and conducted on humans in the 6 countries of GCC. These databases include i) MEDLINE/PubMed, ii) EMBASE, iii) Cochrane Central Register of Controlled Trials (CENTRAL), iv) ClinicalTrials.gov, and v) World Health Organization International Clinical Trials Registry Platform. All trials from World Health Organization International Clinical Trials Registry Platform had been discarded due to incomplete records obtained. The search strategy is demonstrated in **Appendix A**. The 2 reviewers independently extracted information. All the disagreements were resolved by discussion.

### Assessment of risk of bias in included studies

The 2 reviewers independently assessed each eligible article in terms of its risk of bias using the CCRBT, following the criteria of 7 CCRBT domains, namely, i) random sequence generation, ii) allocation concealment, iii) selective reporting, iv) other sources of bias, v) blinding of participants and personnel, vi) blinding of outcome assessment, and vii) incomplete outcome data. We categorized each study for every domain by classifying each as “high risk”, “unclear risk”, or “low risk” of bias with respect to the criteria indicated in the CCRBT. “High risk” of bias means that the bias greatly contributes to the alteration of results.^8,9^ “Unclear” risk of bias implies that inadequate information is presented thereby raising some doubts with regards to the results. Meanwhile, “low risk” of bias indicates no presence of bias or unlikely alteration of results in case a bias is present. We used the term “adequate” and “low-risk” interchangeably. We evaluated the overall risk of bias for every study using the following criteria: “high risk trial”, if at least one domain is classified as “high risk” of bias, “low risk trial” if all domains are found to be of “low risk” of bias, and “unclear risk trial” if all domains contained combination of low and unclear risk of biases.

### Statistical analysis

We encoded and analyzed the collected data using SPSS statistical software package, version 23 (IBM Corp., Armonk, NY). Categorical variables were presented as frequency distributions using descriptive statistics. Data of each CCRBT domain results as well as the overall risk of bias resluts are presented in frequency and percentage. Chi-squared test or Fisher’s exact test was used to test for any possible association between the methodological quality and open access publication, as appropriate. To identify any potential correlation between the quality of RCTs and journal impact factor, Spearman’s correlation test was used, in which “high-risk”, “unclear-risk”, and “low-risk” quality were coded as 0, 1, and 2, respectively. A histogram and linear curve for the risk-of-bias were plotted against publication year, and the results were tested using a non-parametric trend test. A *p*-value <0.05 was considered to be statistically significant.

## Results

**Figure 1:**
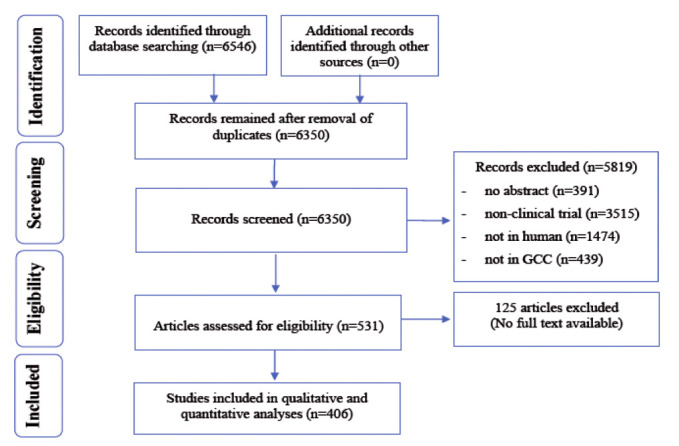
- Flow diagram of study selection.

We identified a total of 6546 articles through database searching, narrowing down the number to 6350 after duplicates were removed. Out of 6350 records, 5819 were excluded, after further screening, yielding 531 articles eligible for the systemic review. Only 406 articles were analyzed out of records for reason that the excluded 125 had no available full text or cannot be found despite exerting effort to retrieve it. Figure 1 shows the selection process of studies.

### Characteristics of randomized-controlled trials (RCTs)

**Figure 2:**
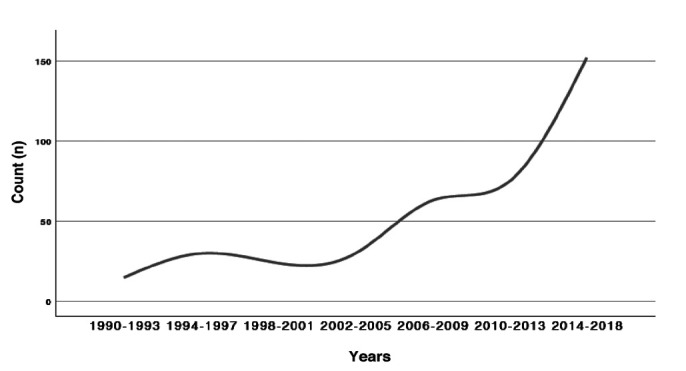
- Number of randomized-controlled trials published between 1990 and 2018.

The number of RCT publications was observed to increase over the period. It increased by 100% from 1900-1993 period to the period of 1994-1997. Likewise, the number of publications in the latest period (2014-2018) was at more than twice that of the preceding period (2010-2013)(Figure 2). In terms of journal section descriptions (Table 1), we found out that the impact factor (2018 - 2019) of the studied articles have a mean value (SD) of 3.54 (6.9). Majority of the studied articles reported no section of declaration of interest (59.1%, n=240), no funding sources (n=263, 64.8%), and were not published in open access journals (67.5%, n=274). For the demographic characteristics (Table 2), we found out that the sample size (n=404) of the studied trials have a mean value (SD) of 201.96 (639.1). Table 3 demonstrates that most of the trials had samples acquired from hospital or clinic (92.9%¸ n=377), no registered protocol (78.8%, n=320), were single-centered (87.2%, n=354), were related to medicine (78.6%, n=319), involved no use of randomization ratio (90.9%, n=369), applied the parallel type of design (87.7%, n=357), and no rate of loss-to-follow up reported (80.8%, n=329). Nearly three-fourth of the studies involved the use of intention-to-treat (ITT) principle (71.8%, n=292). The mean age of participants enrolled in the trials (in years) was 33.83±18.0 (Table 2).

**Table 1:** - Journal-related descriptions of randomized-controlled trials in countries* under Gulf Cooperation Councils from 1990 to 2018 (n=406).

Characteristics	n	Min	Max	Mean	SD
Impact factor of journal (2018-2019)	385	0.06	70.76	3.54	6.9
				n	%
Impact factor of journal (2018-2019)	<1			66	16.3
	1-10			302	74.4
	>10			16	3.9
	NA			22	5.4
Declaration of interest	***Yes***				
	No conflict/competing interest			154	37.9
	Interest mentioned			12	3
	No			240	59.1
Type of funding source	None			263	64.8
	Academic-based			70	17.2
	Government/National Council			23	5.7
	Private			24	5.9
	Hospital-based			20	4.92
	International council/agency			5	1.23
	Non-government/non-profit organization			1	0.25
Publication in open access journals	Yes			112	27.6
	No			274	67.5
	NA			20	4.9

*Saudi Arabia, Bahrain, United Arab Emirates, Kuwait, Oman and Qatar, NA: not applicable, SD: standard deviation

**Table 2:** - Demographic characteristics of randomized-controlled trials in countries* under Gulf Cooperation Councils (GCC) 1990 to 2018 (n=406).

Characteristics	n	Min	Max	Mean	SD
Sample size	404	10	10600	201.96	639.1
Overall mean age (in year)	322	0.54	77.00	33.83	18.0
Overall Proportion of females	292	0.42%	100.0%	46.62%	29.5%
				n	%
Sample source	General population			1	0.2
	Hospital/clinics			377	92.9
	Others			28	6.9
Geographical location	Saudi Arabia			288	70.9
	Kuwait			58	14.3
	Bahrain			2	0.5
	UAE			25	6.1
	Oman			6	1.5
	Qatar			21	5.2
	Collaboration within GCC countries			6	1.5
Registered protocol	Yes			86	21.2
	No			320	78.8
Number of study centers	Single center			354	87.2
	***Multi-center***				
	2 - 25			39	9.6
	>25			4	1.0
	Not numbered			9	2.2
Field of study	Medicine			319	78.6
	Dentistry			52	12.8
	Infectious disease			13	3.2
	Oncology			11	2.7
	Other			11	2.7

*Saudi Arabia, Bahrain, United Arab of Emirates, Kuwait, Oman, and Qatar, SD: standard deviation

**Table 3:** - Methodology of randomized-controlled trials in countries* under Gulf Cooperation Councils from 1990 to 2018 (n=406).

Characteristics	n	%
***Randomization ratio***		
None	369	90.9
1:1	30	7.4
1:1:1	2	0.5
1:1:1:1	1	0.2
2:1	1	0.2
2:2:1	1	0.2
3:1	1	0.2
5:1	1	0.2
***Use of block randomization***		
No	369	90.9
Yes		
Single-block size	16	3.9
Multi-block size	4	0.9
Not disclosed block size	17	4.3
***Type of design***		
Parallel	357	87.7
Cross-over	31	7.6
Cluster-based	7	1.7
Single group assignment	6	1.5
Split-mouth	4	1.0
Split-face	1	0.2
***Intention-to-treat (ITT) and per protocol (PP) analyses***		
ITT	292	71.8
PP	96	23.7
ITT and PP	8	2.0
NA	10	2.5
***Reported overall rate of loss-to-follow up***		
Yes	69	17.0
No	329	80.8
NA	8	2.2

*Saudi Arabia, Bahrain, United Arab of Emirates, Kuwait, Oman, and Qatar, NA: not applicable, ITT: intention-to-treat analysis, PP: per protocol analysis

### Risk of bias in the included studies

Results of risk of bias assessment of included studies are summarized in the following subsections.

### Random sequence generation and allocation concealment (selection bias)

Using CCRBT, we found out that 47.3% (n=192) had low risk while 38.4% (n=156) exhibited unclear risk, and 14.3% (n=58) had high risk of bias for random sequence generation domain. With regard to allocation concealment domain, 55.9% (n=227) of the trials had unclear risk of bias, 24.9% (n=101) exhibited “low risk” of bias, and 19.2% (n=78) showed high risk of bias (Table 4).

**Table 4:** - Overall assessment of risk of bias using Cochrane Collaboration Risk of Bias Tool of randomized-controlled trials in countries* under Gulf Cooperation Councils from 1990 to 2018 (n=406).

Variables	n	%
***Random sequence generation***		
Unclear risk	156	38.4
Low risk	192	47.3
High risk	58	14.3
***Allocation concealment***		
Unclear risk	227	55.9
Low risk	101	24.9
High risk	78	19.2
***Selective reporting***		
Unclear risk	76	18.7
Low risk	328	80.8
High risk	2	0.5
***Other sources of bias***		
Unclear risk	320	78.8
Low risk	60	14.8
High risk	26	6.4
***Blinding of participants and personnel***		
Unclear risk	89	21.9
Low risk	141	34.7
High risk	176	43.4
***Blinding of outcome assessment***		
Unclear risk	172	42.3
Low risk	131	32.3
High risk	103	25.4
***Incomplete outcome data***		
Unclear risk	26	6.4
Low risk	340	83.7
High risk	40	9.9
***Overall risk of bias***		
Unclear risk	185	45.6
High risk	221	54.4

*Saudi Arabia, Bahrain, United Arab of Emirates, Kuwait, Oman, and Qatar

### Blinding (performance and detection bias)

Table 4 shows that 43.4% (n=176) of articles had high risk of bias while 34.7% (n=141) involved low risk of bias in the blinding of participants and personnel domain. The result of blinding of outcome assessment showed that 25.4% (n=103) of studies found to have high risk of bias while 32.3% (n=131) of them exhibited bias of low risk.

### Incomplete outcome data (attrition bias)

We found out that 83.7% (n=340) of trials exhibited low risk of bias while high risk of bias was reported as 9.9% (n=40) (Table 4).

### Selective reporting (reporting bias)

Majority of trials had low risk of bias (80.8%, n=328) while high risk of bias was accounted as 0.5% (n=2) (Table 4).

### Other potential sources of bias

We found out that most of the trials had unclear risk of bias (78.8%, n=320), followed by low risk (14.8%, n=60) and high risk of bias (6.4%, n=26), with respect to other potential sources of bias (Table 4).

### Trends of the randomized-controlled trial quality

**Figure 3:**
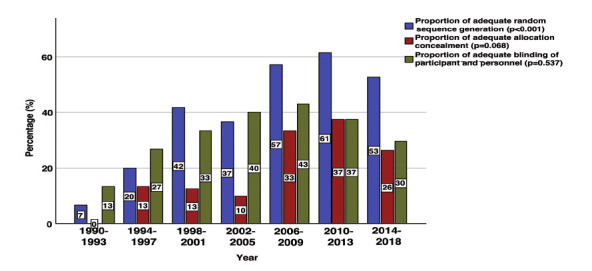
- Quality of randomized-controlled trials published between 1990 and 2018.

**Figure 4:**
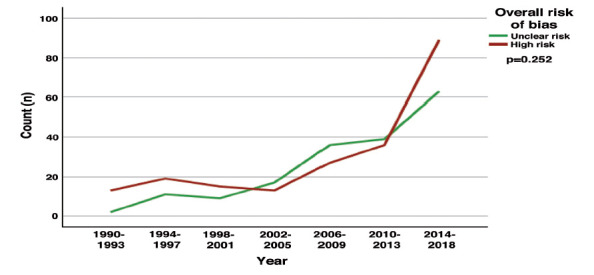
- Trend of overall risk of bias of randomized-controlled trials based on Cochrane Collaboration Risk of Bias Tool between 1990–2018 years.

Apart from a slight decline in the latest period, the proportion of adequate (low risk) random sequence generation methods of RCTs increased significantly over the studied period (*p*<0.001). Similarly, the rate of adequate allocation concealment and blinding increased throughout the same period; however, this increase was not statistically significant (*p*>0.05; Figure 3). Concerning the overall risk of bias of RCTs published from 1990 through 2018, an upward trend in the number of studies with unclear and high-risk quality was found. The number of RCTs with high-risk quality increased more markedly in the last period (2014–2018) compared with the number of RCTs with unclear risk of bias; however, the difference did not achieve a statistically significant level (*p*=0.252; Figure 4). None of the included RCTs were deemed low risk in the overall risk of bias domain.

### Associations of the randomized-controlled trial quality with open access publication and journal impact factor

Table 5 delineates the relationship of selected quality domains (random sequence generation, allocation concealment, blinding of participants and personnel, and overall risk of bias) with open access publication and journal impact factor for 2 distinct periods (1990-2005 and 2006-2018). No statistically significant differences between the quality of RCTs and open access publication (*p*>0.05) were found in either period. The random sequence generation domain exhibited a statistically significant correlation with journal impact factor, in the latter half of the studied period (2006-2018); a positive correlation was identified between quality of randomly generated sequence methods and the journal impact factor (r=0.145; *p*=0.014).

**Table 5:** - Association of quality of randomized-controlled trials (RCTs) with journal impact factor (2018-2019) and open access publication (n=406).

Variable	1990-2005			2006-2018		
	Open access publication		JIF R (*P*-value)	Open access publication		JIF R (*P*-value)
	Yes n (%)	No n (%)		Yes n (%)	No n (%)	
***Random sequence generation***						
Unclear risk	9 (69.2)	43 (50.0)	0.141 (0.166)	31 (31.3)	58 (30.9)	0.145 (0.014)*
Low risk	2 (15.4)	26 (30.2)		55 (55.6)	107 (56.9)	
High risk	2 (15.4)	17 (19.8)		13 (13.1)	23 (12.2)	
*P*-value	0.453			0.952		
***Allocation concealment***						
Unclear risk	9 (69.2)	60 (69.8)	−0.003 (0.980)	46 (46.5)	95 (50.5)	0.096 (0.107)
Low risk	1 (7.7)	9 (10.5)		32 (32.3)	57 (30.3)	
High risk	3 (23.1)	17 (19.8)		21 (21.2)	36 (19.2)	
*P*-value	1.000			0.810		
***Blinding of participants and personnel***						
Unclear risk	4 (30.8)	18 (20.9)	0.079 (0.440)	21 (21.2)	45 (23.9)	0.073 (0.217)
Low risk	1 (7.7)	29 (33.7)		37 (37.4)	61 (32.5)	
High risk	8 (61.5)	39 (45.4)		41 (41.4)	82 (43.6)	
*P*-value	0.162			0.699		
***Overall risk of bias***						
Unclear risk	2 (15.4)	37 (43.0)	0.057 (0.577)	47 (47.5)	91 (48.4)	0.095 (0.110)
High risk	11 (84.6)	49 (57.0)		52 (52.5)	97 (51.6)	
*P*-value	0.071			0.902		

*Statistically significant (*p*<0.05), JIF: Journal impact factor, R: Spearman’s rho (spearman correlation coefficient)

## Discussion

### Summary of main results

Our review highlighted the trend of numbers of publications from the targeted countries. There was a remarkable shift in numbers of publications from 2006 onwards. The largest number of publications were from Saudi Arabia and were hospitals/clinics-based studies. Females were slightly less across the trials (46.62%) in comparison to males. There was a lack of randomization in most reports, and nearly three-fourth of the studies involved the use of ITT principle. However, the proportion of adequate random sequence generation methods increased remarkably over the studied period and was found to be positively correlated with the journal impact factor.

### Number of RCTs over the studied period

The result of the present study showed an increasing number of RCTs over the recent three decades. Similar trend was observed in the systematic review of Rajab and colleagues wherein RCTs from Saudi Arabia were shown to increase in number from 1996 to 2018.^9^ This is also in agreement with the results of a review by Chung and Lee^1^ at Korean Medical Institution and by Chung et al^14^ on RCTs published from 1980 to 2005 in the Korean Journal of Family Medicine. The increasing number of RCTs is said to be possibly a product of developing evidence-based medicine.^1,15^

### Quality of the evidence

Research indicates that the lack of randomization, allocation concealment, or blinding in RCTs exaggerates the effect estimate of treatment to various extents. For instance, trials with lack of adequate blinding or allocation concealment exhibit corresponding inflated effect estimates by 17% to 25% and 30% to 41%.^16-20^ The large sample size in this study provides a way to obtain sufficient information for overall assessment of biases related to selection, performance and detection, reporting, attrition and other potential sources in GCC trials. We determined that unclear to high risk of bias is largely present in RCTs conducted in countries of GCC. We determined the overall risk of bias to be of “high risk” (54.4%), while 45.6% had unclear risk and there was none was deemed “low risk”. Evidence on presence of unclear and high risk of biases in the analyzed studies suggests the need for strict attention in designing, conducting and preparing detailed and clear studies to increase the reproducibility and validity of the methods employed as well as the accuracy, validity and reliability of the results.^9,21^

Overall, this study highlighted the use of the CCRBT to identify RCTs exhibiting high risk of biases that could affect the accuracy, validity and reliability of its results. The CCRBT is widely used reliable tool for assessment of risk of bias since it involves transparency in reporting.^22^ Though it has limitations of possibly consuming too much time when employed to assess a trial in comparison to other scale such as Jadad scale.^21^

With regards to quality analysis of RCTs using CCRBT, the result of the current study shows that low number (14.3%) of the analyzed trials had high risk of bias in the random sequence generation domain. This is consistent with the report of Rajab and colleagues in 2019 wherein low number of trials (<2%), was categorized as “high risk” of bias in the said domain.^9^ Also, “unclear risk” of bias was accounted to be 38.4% in the present study for the mentioned domain. This result is relatively higher compared to the report of Alfahmi et al^23^ wherein roughly one-fourth (25.9%) of 27 RCTs, assessed using the 2010 Consolidated Standards of Reporting Trials (CONSORT) statement, had unclear or no randomization. Contrary to this, the result of the current work is relatively lower compared to the report of Saquib et al^7^ (50%) on Saudi Arabian RCTs on behavioral modification, and also to the review of 307 RCTs conducted and published in China last 2004 by Zhang et al^24^ concerning the failure of reporting the randomization method. The proportion of reports with adequate random sequence generation methods in the Gulf region has increased yearly, suggesting that more attention has been paid to the randomization aspect of quality over the last 3 decades.

When it comes to allocation concealment domain, majority of the trials exhibited unclear risk of bias (55.9%). Rajab et al^9^ reported less proportion of “unclear” risk of bias (44%) in the said domain in their analyzed Saudi Arabian RCTs. In contrast, the result of the present study is in agreement with the report of Alfahmi et al^23^ in which unclear allocation concealment was reported for the majority (88.9%) of the trials evaluated using the 2010 CONSORT statement. Saquib et al^7^ also found out that 69% of the 16 Arab Gulf States RCTs assessed by Jadad Scale and CCRBT provided no explanation on how allocation concealment was implemented, suggesting the need for improvement in illustrating allocation concealment in RCTs. Schulz et al^25^ emphasized the importance of incorporating allocation concealment into a study to avoid any influence in randomization and blinding methods and to prevent distortions in the results. Our review reveals that, among the selected quality parameters, allocation concealment was the least to show adequacy throughout the 3-decade period. Similar to the blinding domain, the proportion of adequate allocation concealment has remained less than 50% over the years.

For the blinding of participants and personnel domain, 65.3% of the trials suffered from unclear to high risk of biases combined. This is comparable with the report of Rajab et al^9^ in which their studied trials exhibited 54.1% of unclear to high risk of biases combined. On the other hand, the result of blinding of outcome assessment revealed that 25.4% of the analyzed trials exhibited high risk of bias, which is roughly twice as high as the results (13.1%) reported by Rajabet al.^9^ The result of the current study is relatively in comparable (high and unclear risk combined, 65.3%) with the review of Zhang et al^24^ wherein a high rate of RCTs (82.7%) in China provided no information on blinding of either participants or investigators, as well as comparable with the report of Saquib et al^7^ in terms of the blinding of outcome assessment. It is evident that inconsistent treatments potentially are provided, participant may behave in a different way, or the outcome is not measured objectively, if the blinding element was violated.^24,26^

The overall risk of bias revealed that 54.4% of the trials was of “high risk” category which is comparable with the result of risk of bias analysis in Saudi Arabian RCTs conducted by Saquib et al.^7^ Also, both the results of the current study and of Rajab et al^9^ revealed that none of the RCTs exhibited “low risk” in the overall assessment. Other studies conducted by Chung et al^14^ and Chung and Lee^1^ in Korea reported a relatively similar result concerning a very low number to zero RCTs categorized as “low risk” of bias according to CCRBT assessment.

### Study limitations

We identified some studies possibly relevant to our review; however, their full-text were not available. Another limitation is that the search was performed in only a certain number of databases. Finally, our results are potentially affected by how the original study was reported, such as quality of reporting.

### Implications of findings for future research

Research from GCC countries has been gaining momentum with its high-quality designed methods. This promising trajectory opens up avenues for more reliable healthcare-related evidence from this developing nation. Meanwhile, readers should be mindful of the quality assessment and be discerning in adopting the current evidence.

In conclusion, the study provides reflections on the quality of RCTs published from the GCC countries in the past 3 decades. The proportion of adequately generated random sequence methods has increased remarkably over the 3-decade period, indicating that the randomization methods have gained more attention. Our study also highlighted some important parameters including journal-related measures, of which journal impact factor is positively correlated with the randomization quality. Therefore, journal impact factor can be an added indicator of adequate randomization quality. The large rate of overall high risk of bias in GCC studies necessitates that future researcher to attach more importance to the metrological quality of RCT by ensuring adequacy in the allocation concealment and blinding methods. Our review addresses the gap in which the result may be useful as reference for future researchers in the said area to work on detailed and meticulously designed studies addressing communicable and non-communicable diseases, and indirectly save future unwanted medical expenses.

## Appendix

**Appendix 1:** - Sources, search strategy, and number of records in the review.

Source	Search period	Search strategy	Number of records
Medline	01/01/1990 to 06/29/2018	Syntax used by Hayenes et al. (https://www.ncbi.nlm.nih.gov/books/NBK3827/#pubmedhelp.Clinical_Queries_Filters) For Category: Therapeutic Optimized for: sensitive/broad Sensitive/specific: 99%/70% (clinical[Title/Abstract] AND trial[Title/Abstract]) OR clinical trials as topic[MeSH Terms] OR clinical trial[Publication Type] OR random*[Title/Abstract] OR random allocation[MeSH Terms] OR therapeutic use[MeSH Subheading]).	3469
EMBASE	01/01/1990 to 06/29/2018	(clinical [Title/Abstract] AND trial[Title/Abstract]) OR clinical trials as topic[MeSH Terms] OR clinical trial[Publication Type] OR random*[Title/Abstract] OR random allocation[MeSH Terms] OR therapeutic use[MeSH Subheading]).	1993
Cochrane Central Register of Controlled Trials (CENTRAL)	01/01/1990 to 06/29/2018	Search trials, source (“Saudi Arabia” OR “Oman” OR “Kuwait” OR “Qatar” OR “Emirates” OR “Bahrain”), using search limits which included: All databases	773
ClinicalTrials.gov	01/01/1990 to 06/29/2018	Through clinicaltrials.gov, advanced search, status completed, country (gulf countries one by one), phase 3	311
Total			6546 records

## References

[1] Chung JH , Lee SW. Assessing the quality of randomized controlled urological trials conducted by Korean medical institutions. Korean J Urol 2013; 54: 289–296. 23700493 10.4111/kju.2013.54.5.289 PMC3659221

[2] Sackett DL , Rosenberg WM , Gray JA , Haynes RB , Richardson WS. Evidence based medicine: what it is and what it isn’t. BMJ 1996; 312: 71–72. 8555924 10.1136/bmj.312.7023.71 PMC2349778

[3] Uetani K , Nakayama T , Ikai H , Yonemoto N , Moher D. Quality of Reports on Randomized Controlled Trials Conducted in Japan: Evaluation of Adherence to the CONSORT Statement. Intern Med 2009; 48: 307–313. 19252352 10.2169/internalmedicine.48.1358

[4] You YN , Cho MR , Park JH , Park GC , Song MY , Choi JB , et al. Assessing the quality of reports about randomized controlled trials of scalp acupuncture treatment for vascular dementia. Trials 2017; 18: 205. 28464917 10.1186/s13063-017-1945-0 PMC5414371

[5] Lim SM , Shin ES , Lee SH , Seo KH , Jung YM , Jang JE. Tools for assessing quality and risk of bias by levels of evidence. J Korean Med Assoc 2011; 54: 419–429.

[6] Mokdad AH , Jaber S , Aziz MI , AlBuhairan F , AlGhaithi A , AlHamad NM , et al. The state of health in the Arab world, 1990-2010: an analysis of the burden of diseases, injuries, and risk factors. Lancet 2014; 383: 309–320. 24452042 10.1016/S0140-6736(13)62189-3

[7] Saquib N , Ibrahim AY , Saquib J. Behavioral trials in the Arab Gulf States: A scoping review. SAGE Open Med 2019; 7: 2050312119846787. 31041101 10.1177/2050312119846787 PMC6482655

[8] Higgins JPT , Altman DG , Sterne JAC. Assessing risk of bias in included studies. In: Higgins JPT , Churchill R , Chandler J , Cumpton MS , eds. Cochrane Handbook for Systematic Reviews of Intervantions version 5.2.0. Cochrane; 2017.

[9] Rajab AM , Hamza A , Aldairi RK , Alaloush MM , Saquib J , Saquib N. Systematic review on the quality of randomized controlled trials from Saudi Arabia. Contemp Clin Trials Commun 2019; 16: 100441. 31517135 10.1016/j.conctc.2019.100441 PMC6737301

[10] AlKabba AF , Hussein G , Kasule OH , Jarallah J , Alrukban M , Alrashid A. Teaching and evaluation methods of medical ethics in the Saudi public medical colleges: cross-sectional questionnaire study. BMC Med Educ 2013; 13: 1–8. 24020917 10.1186/1472-6920-13-122 PMC3850889

[11] Latif R. Medical and biomedical research productivity from the Kingdom of Saudi Arabia (2008-2012). J Family Community Med 2015; 22: 25–30. 10.4103/2230-8229.149583 PMC4317991 25657608

[12] Al-Bishri J. Evaluation of biomedical research in Saudi Arabia. Saudi Med J 2013; 34: 954–959. 24043009

[13] Page MJ , McKenzie JE , Bossuyt PM , Boutron I , Ho mann TC , Mulrow CD , et al. The PRISMA 2020 statement: an updated guideline for reporting systematic reviews. BMJ 2021; 372: n71. 33782057 10.1136/bmj.n71 PMC8005924

[14] Chung W , Lee KW , Hwang IH , Lee DH , Kim SY. Quality assessment of randomized controlled trials in the Journal of the Korean Academy of Family Medicine. Korean J Fam Med 2009; 30:626–631.

[15] Keech A , Pike R , Granger R , Gebski V. Interpreting the results of a clinical trial. Med J Aust 2007; 186: 318–319. 17371216 10.5694/j.1326-5377.2007.tb00911.x

[16] Schulz KF , Chalmers I , Hayes RJ , Altman DG. Empirical evidence of bias. Dimensions of methodological quality associated with estimates of treatment effects in controlled trials. JAMA 1995; 273: 408–412. 7823387 10.1001/jama.273.5.408

[17] Moher D , Pham B , Jones A , Cook DJ , Jadad AR , Moher M , et al. Does quality of reports of randomized trials affect estimates of intervention efficacy reported in metaanalyses? Lancet 1998; 352: 609–613. 9746022 10.1016/S0140-6736(98)01085-X

[18] Wood L , Egger M , Gluud LL , Schulz KF , Jüni P , Altman DG , et al. Empirical evidence of bias in treatment effect estimates in controlled trials with different interventions and outcomes: meta-epidemiological study. BMJ 2008; 336: 601–605. 18316340 10.1136/bmj.39465.451748.AD PMC2267990

[19] Kunz R , Vist G , Oxman AD. Randomisation to protect against selection bias in healthcare trials. Cochrane Database Syst Rev 2007: MR000012. 17443633 10.1002/14651858.MR000012.pub2

[20] Kunz R , Oxman AD. The unpredictability paradox: rovicw of empirical comparisons of randomized and non-randomized clinical trials. BMJ 1998; 317: 1185–1190. 9794851 10.1136/bmj.317.7167.1185 PMC28700

[21] Morissette K , Tricco AC , Horsley T , Chen MH , Moher D. Blinded versus unblinded assessments of risk of bias in studies included in a systematic review. Cochrane Database Syst Rev 2011; 2011: MR000025. 21901737 10.1002/14651858.MR000025.pub2 PMC7433288

[22] Hartling L , Ospina M , Liang Y , Dryden DM , Hooton N , Krebs Seida J , et al. Risk of bias versus quality assessment of randomised controlled trials: cross sectional study. BMJ 2009; 339: b4012. 19841007 10.1136/bmj.b4012 PMC2764034

[23] Alfahmi H , Aldawood L , Baz B , Elrggal M , Alsharif HY , Alkahtani SA. Quality of methodological reporting of randomized clinical trials of sodium-glucose cotransporter-2 (sglt2) inhibitors. Arch Pharma Pract 2017; 8: 78–81.

[24] Zhang D , Yin P , Freemantle N , Jordan R , Zhong N , Cheng KK. An assessment of the quality of randomised controlled trials conducted in China. Trials 2008; 9: 22. 18435861 10.1186/1745-6215-9-22 PMC2373774

[25] Schulz KF , Grimes DA. Allocation concealment in randomised trials: defending against deciphering. Lancet 2002; 359: 614–618. 11867132 10.1016/S0140-6736(02)07750-4

[26] Prescott RJ , Counsell CE , Gillespie WJ , Grant AM , Russell IT , Kiauka S , et al. Factors that limit the quality, number and progress of randomised controlled trials. Health Technol Assess 1999; 3: 1–143. 10683591

